# Incidence of elevated lipoprotein (a) levels in a large cohort of patients with cardiovascular disease

**DOI:** 10.1007/s11789-017-0087-y

**Published:** 2017-02-22

**Authors:** Frank van Buuren, Dieter Horstkotte, Cornelius Knabbe, Dennis Hinse, Klaus Peter Mellwig

**Affiliations:** 1 Clinic for Cardiology, Herz- und Diabeteszentrum NRW, Ruhr-Universität Bochum, Georgstr. 11, 32545 Bad Oeynhausen, Germany; 2 Insitute for Laboratory and Transfusion Medicine, Herz- und Diabeteszentrum NRW, Ruhr-Universität Bochum, Bad Oeynhausen, Germany

**Keywords:** Lipoprotein (a), Cholesterol, Cardiovascular disease, Epidemiology, Risk stratifcation

## Abstract

**Background:**

Recently it has been demonstrated that elevated lipoprotein (a) (LPA) levels are associated with an increased risk of cardiovascular disease across multiple ethnic groups. However, there is only scanty data about the incidence of elevated LPA levels in different patient cohorts. As a consequence, we aimed to examine whether patients with elevated LPA levels might be seen more often in a cardiovascular center in comparison to the general population.

**Methods:**

We reviewed LPA concentrations of 52,898 consecutive patients who were admitted to our hospital between January 2004 and December 2014. We subdivided them into different groups according to their LPA levels. Data was compared to available information in medical literature.

**Results:**

26.4% of the patients had LPA levels >30 mg/dl which is in line with the data from literature. Mean level of LPA concentration in our study was twice as high in comparison to the general population (25.8% vs. 13.3%). 4.6% had LPA levels >98 mg/dl (general population <0.3%).

**Conclusion:**

In patients admitted to a cardiovascular center the proportion of LPA >30 mg/dl is comparable to the general population but mean levels over all are twice as high and the proportion of patients with LPA levels of >98 mg/dl is extremely higher.

## Background

Total cholesterol, high density lipoprotein (HDL), and low density lipoprotein (LDL) are frequently used to assess the risk of atherosclerosis due to dyslipidemia [1]. In recent years it came evident that lipoprotein (a) (LPA) as well is supposed to play an important role in the genesis of atherosclerosis and thrombosis.

LPA is composed of an LDL-like particle in which apolipoprotein B100 is covalently attached to apolipoprotein (a) by a disulfide bond [2, 3]. Plasma levels of LPA are genetically determined by variation in the LPA gene coding for apolipoprotein (a) [4]. Apolipoprotein (a) has high structural resemblance to plasminogen [5]. Hence, due to its composition, Erqou concluded in a meta-analysis that there is a continuous, independent association of LPA and the risk of coronary heart disease [6]. Furthermore, epidemiological data suggest that elevated LPA levels are pro-atherogenic while the exact molecular mechanism by which LPA contributes to the atherosclerotic process remains unclear [4, 7–10].

The incidence of LPA levels >30 mg/dl is supposed to be between 7 and 26% in a general European population [1, 11–13]. LPA levels >30 mg/dl are proved to be independently associated with a three-fold risk of major adverse cardiovascular events in patients after coronary artery bypass grafting [3]. Other diseases like thromboembolism and chronic heart failure due to impaired left ventricular function are currently evaluated in larger patient groups [8]. Aortic valve stenosis (AVS) is a valvular heart disease of increasing interest as its prevalence rises with age and is estimated to affect up to 2–7% of all individuals aged >65 years [14, 15]. The identification of risk factors for valvular calcifications and AVS is of utmost interest as the burden of AVS is high and often ends up in surgical or catheter-based treatment like transcatheter aortic valve implantation (TAVI) [14, 16]. In the last years it became evident that AVS progression shares risk factors with atherosclerosis. There are also hints that elevated LPA levels contribute to an increased incidence of AVS [8]. In addition, very recent studies report on a relationship between mitral valve stenosis and elevated LPA levels in patients with coexisting peripheral arterial disease [17].

Next to cardiac manifestations it could be demonstrated that elevated LPA levels are associated with extracardiac arteriosclerosis like peripheral arterial disease and stenosis of the arteria carotis [18, 19]. Von Depka reports about elevated LPA levels as an independent risk factor for venous thromboembolism [11]. Furthermore, it has been hypothesized that LPA contributes to initial wound healing but inhibits external fibrinolysis in a later phase of the healing process leading to its corresponding problems [20]. Moreover, a higher risk for abortions was reported in patients with elevated LPA levels [13].

In summary, elevated LPA levels seem to predispose for numerous non-cardiovascular and cardiovascular diseases and all these studies thrust LPA into the limelight as it appears to play an important role in various processes [21]. However, there is only rare data about the dispersion of elevated LPA levels in different patient communities.

As a consequence, we aimed to examine whether patients with elevated LPA levels might be seen more often in a cohort with cardiovascular disease in comparison to a normal population.

## Patients and methods

In a total of 52,898 consecutive patients (37.81% females, age 61.08 ± 11.03 years) admitted to our Center between January 2004 and December 2014 we analyzed their full lipid profile including LPA, triglycerides, total cholesterol, HDL, and LDL. Furthermore, we checked for diabetes mellitus (glycated hemoglobin = HbA1c) and renal function (creatinine, glomerular filtration rate (GFR)). Patients were assigned to seven different groups according to their LPA concentrations (30 mg/dl steps and more than 180 mg/dl).

Table 1 shows the distribution of LPA concentrations in the different groups.

**Table 1:** Laboratory findings in 52,898 patients. Subdivision according to their LPA levels and number of individuals in the different LPA-groups with LDL > 130 mg/dl, HDL < 40 mg/dl, HbA1c > 6.4% or creatinine > 1.29 mg/dl

LPA			LDL		HDL		HbA1c		Crea	
mg/dl	*n*	%	*n* >130 mg/dl	%	*n* <40 mg/dl	%	*n* >6.4%	%	*n* >1.29 mg/dl	%
<180	206	0.39	107	51.94	36	17.48	68	33.01	43	20.87
151–180	379	0.72	173	45.65	76	20.05	125	32.98	97	25.59
121–150	771	1.46	345	44.75	179	23.22	236	30.61	181	23.48
91–120	1635	3.09	696	42.57	365	22.32	535	32.72	315	19.27
61–90	4934	9.33	1930	39.12	1124	22.78	1930	39.12	932	18.89
31–60	6144	11.61	2219	36.12	1741	28.34	2180	35.48	1209	19.68
0–30	38,829	73.40	13127	33.81	10280	26.48	14741	37.96	6993	18.01

*LPA* lipoprotein (a), *LDL* low density lipoprotein, *HDL* high density lipoprotein, *HbA1c* glycated hemoglobin, *Crea* creatinine

## Results

32.9% out of 52,898 patients had LPA levels of >20 mg/dl (26.6% > 30 mg/dl and 18.4% >50 mg/dl correspondingly). There was no difference in the groups according HbA1c levels and renal function (creatinine).

## Discussion

The last three decades have seen favorable trends in LDL hypercholesterolemia most likely due to an increased awareness in the context of cardiovascular disease and the extended use of lipid-lowering drugs [22]. It was demonstrated that increased circulating levels of LPA are associated with an increased risk ofcardiovascular disease (coronary heart disease, aortic valve stenosis etc.) and stroke [23].

A substantial fraction of the general population has LPA concentrations that might place them at increased risk for cardiovascular disease [21]. Unfortunately there is only scanty data about the incidence of increased LPA levels in the general population and, even more interesting, in populations with cardiovascular disease [7].

### Proportion of different elevated LPA levels in the general population

Plasma levels of LPA are similar in men and women. Epidemiological studies showed that LPA concentrations are lowest in non-Hispanic Caucasians (median 12 mg/dl), Chinese (median 11 mg/dl), and Japanese (median 13) [24, 25]. On the other hand, they are supposed to be slightly higher in Hispanics (median 19), and even higher levels can be seen in individuals of African origin (median 39).

The typical distributions of LPA in the general Caucasian population can be derived from the Copenhagen City Heart Study, in which more than 20,000 individuals were included. In a subgroup analysis with 3000 men and 3000 women they found that 20% had LPA plasma levels >50 mg/dl representing individuals above the 80th percentile [24]. This data is in line with our findings, where 18.4% of our individuals turned out to have LPA levels >50 mg/dl.

Other surveys report about the incidence of LPA levels >30 mg/dl in a general adult population in Italy [12]. In that analysis 1195 individuals were screened and about 26% of them had levels >30 mg/dl. This correlates to the proportion of patients in our group in which 26.6% showed corresponding levels. Bucci reports about LPA levels >20 mg/dl in about 25% of a general population of Caucasian origin [26]. In contrast, 32.9% of our patient cohort had LPA levels >20 mg/dl indicating that elevated levels might predispose for admission in a cardiovascular center.

### Mean levels of LPA in different communities

In terms of mean LPA levels the results are markedly different. However, the comparison to available data in literature is difficult as our study includes patients with cardiovascular disease and not members of a general population.

Hopewell investigated the impact of elevated LPA levels on coronary heart disease and compared his findings with the LPA levels of a control group which was supposed to be healthy [27]. Mean age in this group was 60.9 years, mean LPA levels in the healthy individuals were 10.56 mg/dl. In a general population study, Langsted found mean LPA levels of 17.3 mg/dl [28] compared tos 13.3 mg/dl in a survey performed by Bucci [26]. In contrast, mean levels were twice as high in our patient collective (25.8 mg/dl). This might support the hypothesis that elevated LPA levels can predispose for cardiovascular diseases.

### Extremely elevated LPA levels

The highest levels of LPA in the different general population groups were between 98 and 217 mg/dl [26, 29]. However, these extreme concentrations were rare and in none of the surveys higher than 1%. Our population included 2415 individuals (4.6%) with levels >98 mg/dl and 586 with levels >150 mg/dl (1.1%). These findings suggest that patients who were admitted to a cardiovascular center more often have extremely high levels of LPA.

Up to now we cannot provide the exact diagnosis of the patients with extreme LPA levels as this is still subject of an ongoing analysis.

### Treatment options

Up to now elevated LPA concentrations are largely resistant to therapeutic interventions like drug therapy or diet and thus the appropriate strategy in terms of acceptable LPA levels and coexisting risk factors has been discussed extensively. Due to their genetic disposition, LPA levels are intraindividually stable over time.

Niacin has a potential to decrease plasma LPA levels by approximately 20–30% [21]. However, it was withdrawn from the market due to major side effects, mainly hepatotoxicity and flushing.. Furthermore, two recent clinical trials found that, despite positive effects on LPA plasma levels, niacin failed to improve clinical outcome endpoints [30]. In particular there is no data that describes the influence of niacin therapy on LPA levels in a large community.

Alternatively, newly developed drugs like mipomersin, a second generation antisense oligonucleotide, reduces plasma levels of LPA by 21–36% [31]. Moreover, PCSK9-inhibitors, drugs that affect LPA levels by influencing the LDL–receptor degradation, may reduce LPA levels by up to 30%. However, neither for this therapeutic option there are still no data available illuminating clinical endpoints [32].

Currently, only LPA apheresis is a proved therapy to reduce LPA levels by more than 60% per session thus having a positive influence on the reduction of cardiovascular events [33–37].

## Conclusion

The presented data shows, as far as we know, for the first time the incidence of elevated LPA concentrations in a large cohort of patients in a cardiovascular center. Mean LPA concentrations are higher and extreme LPA values are seen more often in cardiovascular patients whereas the proportion of individuals with levels >30/mg is similar in comparison to the general population. Against this background the crucial questions of correct cut-off values and the new therapeutic options beyond apheresis therapy have to be addressed in the upcoming years.

## Limitations of the study

The study was not designed as a population-based study because all patients are individuals who were admitted to a cardiovascular center. Many of them were diagnosed to suffer from cardiac disease. However, the proportion of patient in whom no relevant cardiovascular disease was found is unknown and is still subject of ongoing analysis.
